# The Brain Economy: Advancing Brain Science to Better Understand the Modern Economy

**DOI:** 10.21315/mjms2024.31.1.1

**Published:** 2024-01-15

**Authors:** Harris A. Eyre, William Hynes, Rym Ayadi, Pawel Swieboda, Michael Berk, Agustin Ibanez, María E. Castelló, Dilip V. Jeste, Michelle Tempest, Jafri Malin Abdullah, Kelly O’Brien, Steve Carnevale, Alfred K. Njamnshi, Michael Martino, Dan Mannix, Katrina Maestri, Ruojuan YU, Shuo CHEN, Chee H. NG, Heinrich C. Volmink, Rajiv Ahuja, Frederic Destrebecq, George Vradenburg, Astrid Schmied, Facundo Manes, Michael L. Platt

**Affiliations:** 1 Brain Capital Alliance, San Francisco, California, USA; 2 Center for Health and Biosciences, The Baker Institute for Public Policy, Rice University, Houston, Texas; 3 Meadows Mental Health Policy Institute, Dallas, Texas, USA; 4 Euro-Mediterranean Economists Association, Barcelona, Spain; 5 Institute for Mental and Physical Health and Clinical Translation (IMPACT), Deakin University and Barwon Health, Geelong, Victoria, Australia; 6 Department of Psychiatry and Behavioral Sciences, Baylor College of Medicine, Houston, Texas, USA; 7 Department of Psychiatry and Behavioral Sciences, University of Texas Health Sciences Center, Houston, Texas, USA; 8 Global Brain Health Institute, University of California, San Francisco (UCSF), San Francisco, California and Trinity College Dublin, Dublin, Ireland; 9 FondaMental Fondation, Paris, France; 10 Latin American Brain Health Institute (BrainLat), Universidad Adolfo Ibáñez, Santiago de Chile, Chile; 11 Houston Methodist Behavioral Health, Houston Methodist Academic Institute, Houston, Texas, USA; 12 Department of Psychiatry and Behavioral Sciences, University of California, California, USA; 13 Frontier Technology Lab, School of Engineering and Doerr School of Sustainability, Stanford University, California, USA; 14 Rebuilding Macroeconomics, University College London, London, United Kingdom; 15 Santa Fe Institute, Santa Fe, New Mexico, USA; 16 School of Advanced International Studies Europe, Johns Hopkins University, Bologna, Italy; 17 Bayes Business School, City College London, London, United Kingdom; 18 Center for European Policy Studies, Brussels, Belgium; 19 NeuroCentury, Brussels, Belgium; 20 European Policy Centre, Brussels, Belgium; 21 International Center for Future Generations, Brussels, Belgium; 22 Orygen, The National Centre of Excellence in Youth Mental Health, Centre for Youth Mental Health, Florey Institute for Neuroscience and Mental Health and the Department of Psychiatry, The University of Melbourne, Melbourne, Australia; 23 Laboratorio Interdisciplinario del Tiempo, Universidad de San Andrés-CONICET, Buenos Aires, Argentina; 24 Desarrollo y Evolución Neural, Departamento Neurociencias Integrativas y Computacionales, Instituto de Investigaciones Biológicas Clemente Estable (MEC), Montevideo, Uruguay; 25 Programa de Desarrollo de las Ciencias Básicas (MEC-UdelaR), Montevideo, Uruguay; 26 Fibras, Montevideo, Uruguay; 27 Global Research Network on Social Determinants of Health and Exposomics, La Jolla, California, USA; 28 Candesic, London, United Kingdom; 29 Fellow, Academy of Sciences Malaysia, Menara Matrade, Kuala Lumpur, Malaysia; 30 Chairman of Medical and Health Sciences Cluster, The National Council of Professors, Malaysia (MPN), Selangor, Malaysia; 31 Professor of Neurosciences & Senior Consultant Neurosurgeon, Department of Neurosciences & Brain and Behaviour Cluster, School of Medical Sciences/Hospital USM, Universiti Sains Malaysia Health Campus, Kelantan, Malaysia; 32 UsAgainstAlzhiemer’s, Washington DC, USA; 33 Dyslexia Center, UCSF, California, USA; 34 Brain Research Africa Initiative (BRAIN), Geneva, Switzerland & Yaoundé, Cameroon, Africa; 35 Department of Neuroscience, Medical University of South Carolina (MUSC), South Carolina, USA; 36 School of Management, Yale University, Connecticut, USA; 37 Sutardja Center for Entrepreneurship and Technology, College of Engineering, University of California, California, USA; 38 Department of Psychiatry, The Melbourne Clinic and St. Vincent’s Hospital, University of Melbourne, Australia; 39 School of Public Health, Faculty of Health Sciences, University of the Witwatersrand, South Africa, Africa; 40 Division of Health Systems and Public Health, Department of Global Health, Stellenbosch University, South Africa, Africa; 41 Milken Institute, Center for the Future of Aging, California, USA; 42 European Brain Council, Brussels, Belgium; 43 Davos Alzheimer’s Collaborative, Washington DC, USA; 44 Science of Learning in Education Center, Office of Education Research, National Institute of Education, Nanyang Technological University, Singapore; 45 Institute of Cognitive and Translational Neuroscience (INCYT), INECO Foundation, Favaloro University, Buenos Aires, Argentina; 46 Department of Neuroscience, University of Pennsylvania, Philadelphia, PA, USA; 47 Department of Psychology, University of Pennsylvania, Philadelphia, PA, USA; 48 Marketing Department, University of Pennsylvania, Philadelphia, PA, USA; 49 Wharton Neuroscience Initiative, Wharton Business School, University of Pennsylvania, Philadelphia, USA

**Keywords:** brain science, brain health, brain skills, medicine, research, economy, finance, mental health, psychiatry, neuroscience

## Abstract

The coming years are likely to be turbulent due to a myriad of factors or polycrisis, including an escalation in climate extremes, emerging public health threats, weak productivity, increases in global economic instability and further weakening in the integrity of global democracy. These formidable challenges are not exogenous to the economy but are in some cases generated by the system itself. They can be overcome, but only with far-reaching changes to global economics. Our current socio-economic paradigm is insufficient for addressing these complex challenges, let alone sustaining human development, well-being and happiness. To support the flourishing of the global population in the age of polycrisis, we need a novel, person-centred and collective paradigm. The brain economy leverages insights from neuroscience to provide a novel way of centralising the human contribution to the economy, how the economy in turn shapes our lives and positive feedbacks between the two. The brain economy is primarily based on Brain Capital, an economic asset integrating brain health and brain skills, the social, emotional, and the diversity of cognitive brain resources of individuals and communities. People with healthy brains are essential to navigate increasingly complex systems. Policies and investments that improve brain health and hence citizens’ cognitive functions and boost brain performance can increase productivity, stimulate greater creativity and economic dynamism, utilise often underdeveloped intellectual resources, afford social cohesion, and create a more resilient, adaptable and sustainability-engaged population.

## Introduction

The term ‘knowledge economy’ was first coined in the 1960s by Peter Drucker. A knowledge economy is a system where goods and services are produced based on knowledge-intensive activities. It is an economic system that relies more on intellectual property than natural resources or tangible assets.

Much has changed since the 1960s, however. Today, the knowledge economy is increasingly driven by generative artificial intelligence (AI)-based knowledge. Further, current economic systems have failed to turn around low productivity growth. Productivity is key to the wealth of nations and their living standards (1). With higher productivity, such problems as budget deficits, poverty reduction, healthcare and the environment become far more manageable. Boosting productivity growth is a fundamental economic challenge.

The brain economy, therefore, offers a new opportunity. It integrates the latest insights from the knowledge economy while making primary Brain Capital and other vital brain science-based social, technology, economic and policy dynamics. These are all new levers to boost productivity.

Brain Capital is gaining traction as a framework that explains why human brains, both individually and collectively, are at the centre of society’s future and prosperity (2). Brain Capital is inherently transdisciplinary, combining academic, policy, corporate, health and education interests, towards a common goal. It aligns and consolidates disparate academic, professional, and business disciplines and provides a basis for integrated systems understanding, policy, investment, economic and coordination (3). We seek to establish Brain Capital as a fundamental national asset, as important as Gross Domestic Product (GDP) and road infrastructure in the eyes of society and politicians.

**Figure 1:**
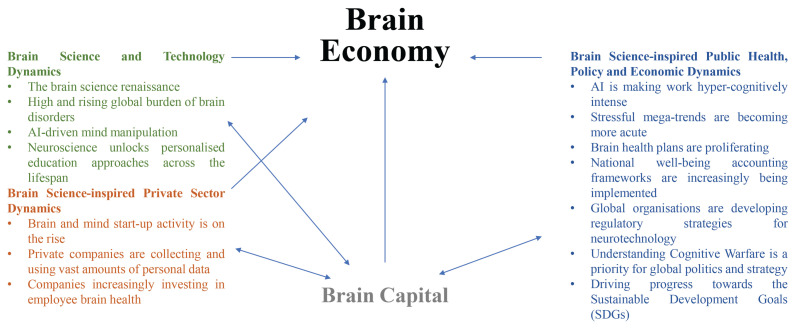
Overview of the brain economy. The brain economy leverages insights from neuroscience to provide a novel person-centred contribution to the economy and how the economy shapes our lives. The brain economy makes primary Brain Capital, an economic framework integrating brain health and brain skills, the social, emotional and the diversity of cognitive brain resources of individuals and communities. The brain economy also integrates other vital brain science-based social, technology, economic and policy dynamics which all interrelate to Brain Capital

In this paper, we provide further reasons why the brain economy is a vital new paradigm spanning brain science-inspired technological, economic and financial innovations. See Figure 1 for a graphical overview.

### Brain Science and Technology Dynamics

#### The Brain Science Renaissance

There are a range of new breakthroughs in neuroscience that unlock new opportunities to understand our brains and minds (4). Neuroimaging, along with preclinical and computational neuroscience studies, has greatly improved our understanding of brain dynamics i.e. patterns of brain activity that are involved in specific traits, cognitive processes or behaviours. Furthermore, a growing evidence-based highlighting the importance of social determinants of health has allowed for more rigorous studies on the brain’s involvement during social interactions, i.e. social neuroscience. Advances in computational methods, including polygenic risk scores and novel techniques (i.e. long reads), are now helping to predict the development of certain psychiatric and neurological conditions, offering novel insights into measures that can prevent brain disorders and slow the progression of disease and cognitive decline. Human neural organoids, transplants, transgenic animal models and chimeras are methodological approaches that also enable the advancement in our understanding of the brain. Finally, technological advancements in machine learning and AI allow us to capture vast data-sets and through analysis with the appropriate computational methods, to uncover novel insights of the human brain and its diseases.

Several of these advancements in brain and mind science were possible through the concerted contribution and collaboration of relevant stakeholders such as neuroscientists, policy makers and research centers within the framework of country, regional and continental brain initiatives (5). Nevertheless, there are still several fundamental questions on brain structure and function that remain elusive, including the mechanisms subserving brain-derived emergent properties such as perception, cognition and behaviour. Extending these innovations and applying these learnings across traditionally underserved communities is also an ongoing priority. In Box 1 we show updates from the Davos Alzheimer’s Collaborative’s (DAC) Malaysia programme. To leverage and capitalise on the brain economy, further public and private investment in brain science is critical.

Box 1: **DAC’s Malaysia programme**

This is the first international cohort to deploy the DAC minimal viable protocol, which exemplifies how DAC tapped ubiquitous research methods to promote brain health. DAC capitalises on the worldwide penetration of tablets and smartphones, along with the collection of blood to collect data that is feasible by anyone, anywhere. Application of deep machine learning and AI analytics to these data will likely accelerate the identification of causes, predispositions and prevention factors that will translate into rapid drug discovery, clinical care and disease prevention. Current estimates indicate that 8.5% of Malaysians aged over 60 years old have dementia, resulting in an estimated dementia population of 260,345.

The conduct of brain health assessments is already underway through a DAC collaborative effort that includes Dr. Rhoda Au and colleagues from Boston University and the research team in Malaysia. DAC anticipates that the study design and learnings can serve as a model across the world, contributing to the overarching goals of building support for the brain economy. One research finding that is likely the first of its kind is voice analysis of a similar test given in U.S. English, Malay, English, Mandarin, Cantonese evidenced high degree of similarity in feature correlation patterns and factor structures—suggesting the potential for a common measure of cognition across multiple languages.

The application of smartphone technology for accurately detecting brain-related changes and predicting Alzheimer’s disease is revolutionising the study of brain health. Unlike traditional neuropsychological assessments, which are limited by cost, participant burden and administration by trained examiners, smartphone applications provide a more flexible and frequent monitoring option that people can do on their own and in their home. This approach offers an effective, cost-efficient and scalable means of detecting significant changes in cognitive and other brain related functions earlier and more accurately during the preclinical neurodegenerative process. To date, Malaysia has collected both digital data and blood from about 535 participants, with plans to try and achieve 1,200 participants over the next year.

#### High and Rising Global Burden of Brain Disorders

Brain disorders, including mental illness and neurologic conditions like dementia and stroke, account for more than 15% of all health-related disability worldwide—more than either cardiovascular disease or cancer. This comes at a huge cost to healthcare systems and society, according to an analysis of data from the most recent Global Burden of Disease (GBD) study presented recently at the Congress of the European Academy of Neurology (EAN 2023) (6). Data from the Brain Health Atlas, powered by the Institute for Health Metrics and Evaluation, suggest this burden will increase in coming years, which is clearly unsustainable to the global economy given the yearly cost is in the trillions - in effect this will reduce the economic security of nations (7). The universality and ubiquity of these disorders, from psychiatric to neurological conditions (8) and aggravated by the publicly known stress of the COVID-19 pandemic, negatively impacting on two of the more common forms of mental disorders, anxiety and depression, as well as suicide, are some reasons why there is increasing public discussion on these issues.

#### AI is Manipulating Minds on a Massive Scale

Beyond the positive benefits of AI to help us generate new understanding of the human brain for the treatment of brain disorders (9), there are increasing capabilities of AI-based algorithms to negatively influence human minds, communications and behaviour. This is best seen via social media platforms, whose use, at times, is associated with worsening youth mental health (10, 11) and by malign actors who prolifically disseminate mis- and disinformation (particularly wedge political and health misinformation) (12).

Current advanced algorithms are primarily text-based. The technology industry is currently shifting towards real-time voice and photorealistic digital personas that can increasingly look, move and express like real people. These algorithms are thus predicted to become much more effective at influencing humans (13). Given the rapid progress of generative AI, the future internet is bound to be more interactive, for better but also for worse. This is concerning and has led Yuval Noah Harari, a renowned scholar and author, to warn about the ability of AI to hack our human operating system within the digital communication environment (14). We are pleased to note the UN Secretary-General recently established an AI Advisory Body to strengthen international governance of AI.

#### Neuroscience Unlocks Personalised Education Approaches Across the Lifespan

The application of neuroscience in unlocking personalised education approaches across the lifespan is crucial for society as it fosters an inclusive learning environment that respects individual differences and optimises educational outcomes. Moreover, it empowers individuals to reach their full potential, thereby contributing to societal progress and innovation through the cultivation of a diverse, skilled and adaptable workforce. Neuroimaging techniques that have been traditionally used in the medical sector are increasingly being applied to study the neurobiology of learning (15). By combining non-invasive, portable, wireless, and more and more handheld and relative low-cost features, technologies like electroencephalography and functional near-infrared spectroscopy are making possible to monitor learning in real time with increasing ecological validity, i.e. in naturalistic educational settings (16). Educational applications of such technologies include, but are not limited to, describing brain changes in learning via structural and functional measures (17), characterising brain dynamics for typical and atypical learning trajectories (18), identifying biomarkers to support the diagnosis of learning disorders using neuroanatomical variations (19), detecting inter brain-to-brain synchronisation through hyperscanning studies (20) and predicting educational outcomes combining brain parameters and modeling (21).

The introduction of neuroimaging techniques into classrooms has arrived to stay, just like emerging technologies, such as laptops and tablets, became progressively normal a few decades ago and started to reform education. Revealing how lifelong learning occurs in unprecedented details is underway. As this evolving knowledge constantly shapes educational systems, there is need to elaborate on regulations and the associated ethical challenges at the intersection of research, practice and policy.

### Brain Science-inspired Public Health, Policy and Economic Dynamics

#### AI is Making Work Hyper-Cognitively Intense

Rapid advancements in generative AI are set to reshape society, revolutionise industries and change the nature and future of work (22). Estimates by the Brookings Institution (23) suggest that in the US alone, approximately 60% of work tasks are facing medium to high exposure to displacement by AI in the coming decades. Work will therefore become increasingly cognitively intense as workers learn how to integrate AI into their daily practices and refocus on high level conceptual and strategic priorities given that AI can do much repetitive and mundane work (24).

#### Stressful Mega-Trends are Becoming More Acute

While it is true society has made tremendous progress in recent decades (25), the convergent issues of climate change, political instability, wars, mis- and disinformation escalation have led some experts to describe the world as in a ‘polycrisis’ (26). A number of these issues are set to become increasingly acute in the coming years, such as climate change and disinformation increases. These changes will resonate with and amplify the other threats and innovations discussed in this paper. Climate change itself with weather extremes, increased air pollution and plastic pollution are known to undermine brain health (27). A recent study showed large-scale societal dynamics are related to brain structure and function. Lebedev et al. (28) correlated large-scale United Kingdom biobank data over 14 years with the local stock market index (FTSE1000) and residents’ mood. They showed maximal stock market volatility was associated with volumetric measures of affective brain regions. These types of analyses may continue to yield findings between major societal challenges and population level brain dynamics.

#### National Brain Health Plans are Proliferating

To respond to the high and rising burden of brain disorders, many countries are exploring their own nation-wide, systemic brain health plans. For example, the Swiss Brain Health Plan (29) was recently launched with the vision for promoting brain health for all across the entire life course. The five key strategic objectives are: i) to raise awareness; ii) strengthen cross-disciplinary and interprofessional training/educational programmes for healthcare professionals; iii) foster research on brain health determinants and individualised prevention of brain disorders; iv) prioritise a holistic (non-disease-specific), integrated, person-centered public health approach to promote brain health and prevent brain disorders through collaborations across scientific, health care, commercial, societal and governmental stakeholders and insurance providers; v) support, empower and engage patients, caregivers, and patient organisations, and reduce the stigma and discrimination related to brain disorders. Other countries have implemented such plans, including Norway, Finland and Germany. These country-wide brain plans open up opportunities for new clinical pathways and clinical instruments. The Brain Care Score is one such tool, a motivational, multidimensional tool for use in primary care for stroke, dementia and depression prevention and management (30). Recent analyses of this Score from the UK Biobank showed individuals with a higher score had a lower risk of developing dementia or having a stroke later in life. Other innovative clinical approaches include the development of a field of brain medicine (31), preventive neuroscience (32), preventive neuroradiology (33) and other recommendations from the European Task for Brain Health Sciences (34).

#### National Well-being Accounting Frameworks are Increasingly being Implemented

Well-being accounting frameworks have gained prominence as nations seek to measure societal progress beyond GDP. The OECD and World Bank have developed guidelines for these frameworks, emphasising multidimensional aspects of well-being (35). These initiatives reflect a shift towards comprehensive assessment of national well-being. For example, Scotland has developed a comprehensive well-being framework known as the National Performance Framework (NPF) (36). This framework includes ‘increased well-being’ as part of its purpose and combines economic measures with a broader range of well-being measures. It sets out the direction and ambition for Scotland with 11 National Outcomes and 81 National Indicators.

#### Global Organisations are Developing Regulatory Strategies for Neurotechnology

The OECD adopted the ‘Recommendation on Responsible Innovation in Neurotechnology’ in 2019, providing a guide for ethical and legal challenges in neurotechnology (37). In 2021, the Chilean Parliament modified the Constitution and became the first nation in the world to have a constitution in force that explicitly addresses the challenges of emerging neurotechnologies (38). UNESCO held a conference on the Ethics of Neurotechnology in 2023, emphasising the need for a governance framework to harness neurotechnology’s potential and address societal risks. Both organisations stress the importance of responsible innovation and regulatory frameworks in neurotechnology.

#### Understanding Cognitive Warfare is a Priority in Global Politics, Strategy and Security

The North Atlantic Treaty Organization (NATO) notes concerningly that cognitive warfare is a key new ‘battleground’ among nations (39). Specifically, the NATO Strategic Warfare Development Command defines this as ‘the activities conducted in synchronisation with other instruments of power, to affect attitudes and behaviours by influencing, protecting, and/or disrupting individual and group cognitions to gain an advantage’. Think of scrambling the enemy’s sense of reality: rampant disinformation to sow enemy confusion, degrading their mental resilience and provoking them to lose trust in their government and society. Scientists recently explored the connections between misinformation and the epistemic integrity of democracy (40). They noted that democracy relies on a shared body of knowledge among citizens, in particular, trust in elections and reliable expertise to inform policy-relevant debates. In their review, they note how mis- and disinformation campaigns are undermining shared knowledge and they outline ways psychology can contribute to countermeasures, e.g. regulation, media literacy education and inoculation. The vulnerabilities of our cognitive processes are laid bare, raising concerns about the ethical implications of such manipulation. Countries must now take steps to prepare their citizens for such types of mis- and disinformation-heavy informational war (41). This demonstrates the importance of brain health and well-being to the national security of their nations (42).

#### Brain Capital Can Drive Progress Towards the Sustainable Development Goals

The UN published a progress report in July of 2023, in which it warned that the SDGs were ‘in peril’, with a mere 12% of targets on track (43). We recently published a report noting how Brain Capital can contribute to all of these goals through its human-centric, systemic and transformative nature (27). Crucial to being inclusive in brain research is leveraging innovation to drive down costs—so implementation of new brain health applications are accessible in low-resourced settings. Brain Capital provides a lens for dealing with economic strain, productivity slowdown, sustainability, gender equity, creativity shortcomings, mental health and well-being, erosion of the social fabric of communities and the need for more industrial innovation.

### Brain Science-inspired Private Sector Dynamics

#### Brain and Mind Start-up Activity is on the Rise

There is a burgeoning mental health venture capital industry searching for scalable brain health solutions (44). The level of yearly venture capital investment into mental health start-ups went from very minor to around USD5 billion in 2021. Levels have now reduced post-COVID but remain in the multiple billions per year. An example of a mechanism to support the development of this area of innovation is the One Mind Accelerator which provides services, guidance and funding to support founders of early-stage brain health technology start-ups.

The traditional VC model drives profits over health outcomes, such that investments are not likely to generate a future of effective mental health solutions. We, therefore, recently proposed the need for a dedicated public-private industrial innovation strategy to support the sustainable and impact-oriented nature of this field of innovation (43). Industrial innovation policy strategies involve governmental intervention in one or more of the applied research innovation stages, from development to prototyping to production, to further technology innovation. We must consider creating a dedicated technology platform to support early-stage developments, acceleration of start-ups, guaranteed contracts for production to parallel development, flexible contracting mechanisms for rapid procurement, workforce issues, technology certification that assured prompt market entry, and mapping and filling supply chains for rapid production and distribution.

#### Private Companies are Collecting and Using Vast Amounts of Personal Data

The prominent neuroscience lawyer, Nita Farahany PhD (45), has well documented how private companies are collecting and using vast amounts of personal data. Given advances in brain technology, these data have the ability to reduce our cognitive liberty, the right to self-determination over one’s brain and mental experiences. Therefore, the aforementioned responsible innovation and regulatory frameworks for neurotechnology are vital to safeguard neural sovereignty.

#### Private Companies Increasingly Investing in Employee Brain Health

Employers are increasingly focused on the mental health and well-being of employees, with some reports estimating 90% or more of employers investing more in mental health and well-being programmes (46). While some of this new focus, particularly on mental health, is driven by employee demand, employers are increasingly recognising the impact of brain health on workforce productivity, retention and overall business success (47). Several employer initiatives, for example, the Business Collaborative for Brain Health and work done by the international design firm HKS, Inc. (48), are now recognising that a broader focus on brain may be an effective framework to address the skills gap. Topping the World Economic Forum’s list of skills most needed are problem-solving, critical thinking, creativity, analytical reasoning, learning agility, cognitive flexibility and complex problem-solving (49). Taking a broader approach to brain health merges the need for whole-health approaches, effective mental health solutions and a workforce equipped with the cognitive skills for the future economy—building more resilient, prepared human capital.

In Table 1, we outline our recommendations to advance the brain economy movement.

The concept of a brain economy is not just timely but critical for addressing the complex challenges of the 21st century. Traditional paradigms are insufficient as the world struggles with unprecedented socio-economic, democratic and environmental stressors. Integrating Brain Capital into a novel economic framework may have a transformative impact, leveraging intrinsically human resources to foster a more resilient, innovative, empathic and prosperous society. The collaborative efforts of our transdisciplinary group bridging disciplines and sectors are crucial in propelling this movement forward. As we move into 2024, we must continue to invest and capitalise on brain science, develop and test policies and financing strategies, and refine measurement tools to achieve the potential of the brain economy.

We need brain scientists, health care providers (e.g. nurses, social workers, pharmacists, psychologists, counselors and physicians), educators and public health professionals to engage in this movement as they have expertise in brain science and behaviour at the individual and social levels and public policy leaders to effect change. Finally, a sustained focus on building brain skills, particularly in relation to citizenship education, can potentially generate a critical mass of informed citizenry who identify and elect leaders that further champion brain health.

## Figures and Tables

**Table 1:** Recommendations to advance the brain economy

**Investments**
To better unlock understanding and new approaches to managing our brains, we require much more investment in brain science, neurotechnology, start-ups, health and educational systems.
We recommend not just to consider conventional investment approaches which are purely profit-seeking, but also other impact-oriented and longer-term approaches. For example, public-private-philanthropic partnership models are engineered to tackle challenges requiring deep, system-level transformation, which in turn require patience, an understanding of the needs and constraints of a broad set of actors, the ability to bring these actors together around well-defined objectives, the willingness to take a longer-term perspective, the capacity to run a robust day-to-day operation, and an appetite for experimentation. 4P models aligned to the brain economy include the Baycrest Centre for Brain Health and Aging, the Davos Alzhiemer’s Collaborative, the Ontario Brain Institute and the UCSF Dyslexia Center. Such models must be assessed and developed across the lifespan and in health and skill domains.
We also encourage the development of novel industrial innovation strategies, as outlined in text.
Finally, moving forward, we will need longer-term forms of investing and we suggest that impact investors with more ‘patient capital’ (e.g. a 10–20 year time horizon such as public pension funds and sovereign wealth funds) are better suited for effective brain capital investments compared to politicians with a 2-to-5-year time horizon and venture capital’s 5-to-7-year time horizon.
We note the Japanese Government’s MIRAI Moonshot Project has bold ambitions for transformative society enhancing technologies for 2050. Their Goal 9 aims to realise a mentally healthy society by increasing peace of mind and vitality by 2050.

**Policy innovation**
Infusing brain science into public policy is a key component of the transition to the brain economy. If the brain economy is to be comprehensive, we must therefore consider brain science innovation in all policy areas, from healthcare, to nature, to digital, foreign affairs, built environment, sustainability and social services. For example, we have previously extensively profiled the importance of reforms to the industrialised food system which is currently undermining population-level Brain Capital (50).
Continuing to advance the regulatory and responsible innovation neurotechnology frameworks outlined in this paper are vital.
We must also explore the development of some kind of Brain Capital Pact, to enable extensive, international collaboration towards a transformative impact of brain capital. Such a collaboration should be ‘neural’—a constellation of inter-dependent regions, nodes and pathways, bridging and activating between sectors, disciplines, systems and jurisdictions, inc sub-national. Given Brain Capital’s cross-cutting nature, it is ideally placed to serve as a platform for defining the post-UN Sustainable Development Goal (SDG) post-2030 framework. Such a pact must consider cross-government coordination and supporting policies, the role of public-private partnerships in fostering economic prosperity and social wellness, and new models for mental well-being. It must consider the concept of a global parliamentary working group on brain health and the brain-based economy driven by large middle-income countries (India, Indonesia, Brazil and Nigeria).

**Measurement**
Further refinement of the recently released Global Brain Capital Dashboard is key to advancing the brain economy transition (51).
To build the complete Dashboard, novel indicators were extracted from a wide range of data sources under the banner of three pillars: Brain Capital Drivers, Brain Health and Brain Skills. Brain Capital Drivers refers to the factors that boost or impede the accumulation of brain capital throughout the life course. Some of these factors include digitalisation, health services, the natural environment, education and social connections. Brain Health examines the mental and neurological health of the population at scale. The domain chronicles the absence of disorders, as well as different issues throughout the human lifespan (childhood, adolescence and aging-related issues). The Brain Skills domain captures key areas for the accumulation of Brain Capital such as cognitive skills, non-cognitive skills, mental flourishing and mental resilience.
